# Evaluation of two synchronized external surrogates for 4D CT sorting

**DOI:** 10.1120/jacmp.v14i6.4301

**Published:** 2013-11-04

**Authors:** Carri K. Glide‐Hurst, Megan Schwenker Smith, Munther Ajlouni, Indrin J. Chetty

**Affiliations:** ^1^ Department of Radiation Oncology Henry Ford Health Systems Detroit MI 48202 USA

**Keywords:** 4D CT, external surrogate, motion management

## Abstract

The purpose of this study was to quantify the performance and agreement between two different external surrogate acquisition systems: Varian's Real‐Time Position Management (RPM) and Philips Medical Systems' pneumatic bellows, in the context of waveform and 4D CT image analysis. Eight patient displacement curves derived from RPM data were inputted into a motion platform with varying amplitudes (0.5 to 3 cm) and patterns. Simultaneous 4D CT acquisition, with synchronized X‐ray on detection, was performed with the bellows and RPM block placed on the platform. Bellows data were used for online retrospective phase‐based sorting, while RPM data were used for off‐line reconstruction of raw 4D CT data. RPM and bellows breathing curves were resampled, normalized, and analyzed to determine associations between different external surrogates, relative amplitude differences, and system latency. Maximum intensity projection (MIP) images were generated, phantom targets were delineated, and volume differences, overlap index, and Dice similarity coefficient differences were evaluated. A prospective patient study of ten patients was performed and waveforms were evaluated for latency (i.e., absolute time differences) and overall agreement. 4D CT sorting quality and subtraction images were assessed. Near perfect associations between the RPM and bellows‐acquired breathing traces were found (Pearson′sr=0.987−0.999). Target volumes were 200.4±12ccand199.8±12.6cc for RPM and bellows targets, respectively, which was not significantly different (U=33,p>0.05). Negligible centroid variations were observed between bellows and RPM‐contoured MIP targets (largest discrepancy=−0.24±0.31mm in superior‐inferior direction). The maximum volume difference was observed for an RPM target 2.5 cc (1%) less than bellows, yielding the largest difference in centroid displacement (0.9 mm). Strong correlations in bellows and RPM waveforms were observed for all patients (0.947±0.037). Latency between external surrogates was <100ms for phantom and patient data. Negligible differences were observed between MIP, end‐exhale, and end‐inhale phase images for all cases, with delineated RPM and bellows lung volumes demonstrating a mean difference of −0.3±0.51%. Dice similarity coefficients and overlap indices were near unity for phantom target volumes and patient lung volumes. Slight differences were observed in waveform and latency analysis between Philips bellows and Varian's RPM, although these did not translate to differences in image quality or impact delineations. Therefore, the two systems were found to be equivalent external surrogates in the context of 4D CT for treatment planning purposes.

PACS numbers: 87.57.Q‐, 07.07.Df

## I. INTRODUCTION

Many motion compensation techniques have been developed in thoracic radiotherapy, one of which is respiratory correlated CT (or 4D CT).^(1)^ 4D CT inherently provides temporal information for both tumor and organ motion by oversampling CT data at each slice and subsequently sorting them into “phases” using an indicator of respiratory state. However, 4D CT may be prone to reconstruction and sorting artifacts introduced by patients' varied and irregular respiratory patterns during 4D CT acquisition, particularly for lung cancer radiotherapy where patients may present with compromised pulmonary function. These artifacts can lead to discrepancies in target and critical structure delineation, as well as impact 3D dose calculation accuracy. In addition, poor 4D CT reconstruction quality may be detrimental to deformable image registration (DIR) performance, which is an integral component of adaptive radiotherapy to facilitate automated contour delineation^(^ ^2^ ^,^ ^3^ ^)^ and cumulative dose estimation.^(4)^

One important aspect in 4D CT is the selection, and overall quality, of 4D CT sorting mechanism. Ideally, internal tumor motion would be used. However, the tumor is not present in all of the axial slices that would be needed for motion extraction. One study explored implementing multiple internal surrogates, such as the air content, lung area, lung density, and body area for 4D CT sorting, and found strong agreement with external surrogates recorded by the real‐time position management (RPM) system.^(5)^ However, when an irregular breathing pattern was explored, poor correlation was realized. Improved internal‐to‐external associations have been observed when multiple markers or deformed surface images were used as external surrogates,^6^, ^7^, ^8^ although these approaches can be computationally expensive and are not currently incorporated into standard clinical practice.

Currently, external surrogates, such as abdominal motion derived from pressure‐sensitive belts, infrared blocks, or surface images, are often employed to derive the signal for 4D CT sorting. No consensus on the “best” external surrogate system for 4D CT sorting currently exists. Otani et al.^(9)^ compared the agreement of Varian's Real‐Time Position Management (RPM) system, consisting of an infrared camera tracking a reflective box, with Anzai Medical's pressure sensor (a small pneumatic sensor) in a population of ten patients. While the two external surrogates were collected simultaneously, there was one chief difference in the signal that may have been collected in this study: the RPM block was placed directly on the patient's skin between the xiphoid process and umbilicus, while the pressure sensor was placed between an immobilizing body mesh and abdomen. The Otani patient study revealed that, while the acquired breathing data between the two systems were found to be well‐correlated, phase differences between external surrogates, changes in tumor centroid, and variations in tumor shape were observed. It is unknown what role the constricting, immobilizing mesh may have played in these findings, particularly the phase difference aspect. Furthermore, the authors did not explore a controlled phantom setting that may better decouple competing mechanisms, such as internal‐external correlations and amplitude differences.

To date, a well‐designed, controlled study directly comparing two of the most widely used external surrogates — a marker block tracked with an infrared camera (Varian's RPM) and a pneumatic belt (Philips bellows) — has not been explored, particularly with hardware and software that allows for synchronized data acquisition. This work assesses, first in a controlled phantom experiment and later extending to prospective patient analysis, the performance and agreement of the external surrogate systems, image quality, and volumetric/positional changes in reconstructed data. Results from this work can be used to support the selection of external surrogate tailored to each clinic's needs and available equipment.

## II. MATERIALS AND METHODS

### A. External surrogates

Both Real‐Time Position Management Respiratory Gating System (RPM) (Version 1.7.5, Varian Medical Systems, Palo Alto, CA) and the pneumatic belt (bellows) (Philips Medical Systems, Cleveland, OH) track the patient surface (i.e., chest or abdominal wall) and serve as external surrogates for respiration‐induced tumor motion. Briefly, the original RPM system uses a plastic block containing two markers that reflect infrared light. These markers are subsequently tracked with an infrared‐sensitive charge‐coupled device camera, and this video signal is transferred back to the RPM computer. The bellows, on the other hand, consists of a rubber belt that expands and contracts as patients' breathing volumes change. Changes in the pressure are converted via a transducer to a voltage signal, that is then digitized and sent to the CT scanner system.

### B. 4D CT acquisition and waveform generation

All phantom and patient 4D CTs in this study were acquired using multislice helical CT (Brilliance CT v2.3.5; Philips Medical Systems) for retrospective phase‐based sorting into 10 phases (0% representing end‐inhale, while 50% represented end‐exhale). Simultaneous bellows and RPM data acquisition was configured to enable the detection of “X‐RAY ON” in both external surrogate datasets. Briefly, for the RPM system, a LEMO coaxial connector is used to detect the X‐RAY ON indicator arising from transistor‐transistor logic (TTL) from the CT scanner. RPM tracking data, at a frequency of 30 Hz, can then be exported to a VXP text file that includes amplitude, phase, timestamp, and the binary X‐RAY ON indicator. X‐RAY ON indicates when the CT is acquiring data, and this timestamp enables synchronization of the RPM waveform with the CT dataset. For the bellows, an IBOX processor receives the X‐RAY ON information internally and couples this with the bellows data that are received via a RS‐232 serial cable. This coupling — the bellows waveform and X‐RAY ON information — is then sent to the CT reconstruction computer for 4D CT reconstruction.

**Figure 1:**
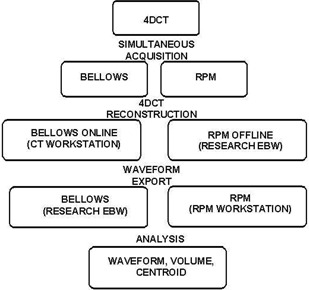
Workflow for simultaneous infrared camera (RPM) and bellows data acquisition used for both phantom and patient experiments. EBW stands for Extended Brilliance Workspace.

Figure 1 shows the workflow for acquisition and subsequent data reduction. For all phantoms and patients studied, bellows data were used for online 4D CT reconstruction using standard clinical acquisition software. A research version of the Extended Brilliance Workspace (Research EBW) (EBW Philips Healthcare, Cleveland, OH) was then used for the following off‐line tasks: (1) to export the bellows waveform from raw 4D CT data for analysis, (2) to retrospectively import the RPM VXP file, and (3) to re‐reconstruct raw 4D CT data using the RPM waveform. The bellows waveform, reported at a frequency of ~38Hz, was exported to a text file containing the displacement (arbitrary units), time, and X‐RAY ON information.

Ten‐phase 4D CT data acquisition involved oversampling the thorax region using a very low couch pitch (typically 0.06–0.08 arbitrary units). Ten equally spaced intervals were generated and the 0% phase was automatically tagged by clinical software (${\text{AcQSim}}^{3}$ CT Simulation Workspace, v3.5, Philips Medical Systems). Modification in automatic tagging occurred in the instance of a missed breathing cycle or improperly tagged end‐inhale peak, which is consistent with our clinical practice. Because of the potential for as‐needed manual intervention, automatic software tagging was not assessed in this study, and the focus was on waveform analysis and image quality differences. Maximum intensity projection images (MIPs) were generated from the maximum intensity value of all voxels over all 4D CT images using ${\text{AcQSim}}^{3}$ for both the RPM and bellows data (termed ${\text{MIP}}_{\text{RPM}}$ and ${\text{MIP}}_{\text{BELLOWS}}$, respectively).

### C. Phantom study

**Figure 2:**
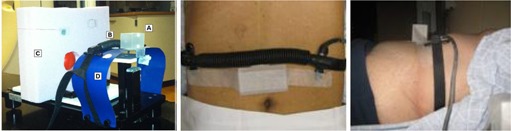
Experimental setup: Left: (A) infrared marker block tracked with the RPM camera; (B) bellows pneumatic belt; (C) lung‐mimicking Styrofoam slab with high‐contrast inserts (D) plastic sheet used to mimic the patient surface. Motion platform translates objects in the superior‐inferior direction, while the marker block and bellows belt translate anteriorposterior on the corresponding chest wall platform. Middle: Overhead view Patient 9, with the bellows superior to the RPM block. Right: Side view of experimental setup for Patient 4.

A series of phantom experiments was performed with a programmable respiratory motion platform (ExacTrac Gating Phantom, Version 1.0, BrainLAB AG, Germany). The platform translated a lung tissue‐mimicking Styrofoam slab with high contrast inserts in the superiorinferior (S‐I) direction, which has been reported as the dominant direction of motion for most lung cancer patients.^(^ ^10^ ^,^ ^11^ ^)^ A corresponding chest wall component moved simultaneously in the anterior‐posterior direction. Both the RPM plastic block and the bellows pneumatic belt were placed on top of a curved plastic sheet, used to provide a surface more representative of patient geometry, affixed to the chest wall platform, as shown in Fig. 2 (left). Bellows and RPM displacement data were acquired simultaneously using the hardware configuration outlined in the Materials & Methods Section B above.

Eight different abdominal (A‐P direction) displacement curves generated from previously acquired patient RPM data were inputted into the motion platform for study. Superior‐inferior object excursion ranged from 0.5 cm to 3 cm, with a wide range of simulated clinical scenarios including baseline drifts, erratic breathing, and varying breathing rates and amplitudes, as demonstrated in Table 1. For each breathing curve studied, in addition to the 4D CTs, three static CT datasets were acquired with the motion platform paused at the following positions: (1) end‐inhale (EI), (2) end‐exhale (EE), and (3) at a midposition (MID). The union of these volumes was expected to represent the “ground truth” of the phantom object volumes for the phantom study, in the absence of any 4D CT sorting artifacts, and was termed ${\text{ITV}}_{\text{TRUE}}$. Deriving ${\text{ITV}}_{\text{TRUE}}$ in this manner assumes equal time is spent at each phantom position, whereas the moving platform combined with helical 4D CT acquisition could introduce uncertainties based on the acquisition start time with respect to breathing irregularities. Therefore, ${\text{ITV}}_{\text{TRUE}}$ yields the maximum phantom volumes that can be expected; nevertheless, it is informative to evaluate the volumes in the absence of artifacts and motion interplay. Subsequent waveform and volume analysis was conducted as described in the Materials & Methods Section E below.

**Table 1:** Measured phantom breathing curve statistics for eight irregular waveforms acquired simultaneously using RPM and bellows

*Phantom*	*External Surrogate*	Amplitude Mean±StDev(A.U.)	*Amplitude %CV*	*Breathing Rate (BPM)*	*Pearson's r* ^a^
1	Bellows	0.146±0.141	96.2	13.9	0.996
	RPM	0.147±0.143	97.3		
2	Bellows	0.095±0.143	150.2	11.9	0.999
	RPM	0.094±0.146	155.1		
3	Bellows	0.307±0.254	83.0	16.9	0.993
	RPM	0.305±0.259	84.8		
4	Bellows	0.171±0.166	97.2	14.9	0.987
	RPM	0.174±0.168	96.5		
5	Bellows	0.206±0.177	85.9	7.2	0.990
	RPM	0.180±0.193	107.4		
6	Bellows	0.271±0.187	69.1	22.0	0.996
	RPM	0.281±0.195	69.3		
7	Bellows	0.218±0.210	96.5	19.9	0.994
	RPM	0.255±0.198	77.8		
8	Bellows	0.110±0.150	136.9	13.0	0.998
	RPM	0.103±0.155	151.1		
				Mean±StDev	0.994±0.004

a All values were statistically significant (p < 0.0001).

### D. Patient study

A prospective nonrandomized Institutional Review Board‐approved study was established at our institution. Between May 2011 and May 2012, 11 patients were enrolled, with one raw 4D CT dataset not recoverable due to operator error, yielding ten patients available for analysis. The inclusion criteria required immobilization that did not interfere with the external monitoring devices and a thoracic lesion of any type. All patients were simulated supine and immobilized in an alpha cradle, with arms extended over head. No abdominal compression was utilized. Patients had varied tumor location, size, and pathology, as demonstrated by Table 2.

For all ten patients studied, both the bellows and marker block placement were placed midway between the xyphoid process and umbilicus. The RPM block was always placed inferior to the bellows to enable proper tracking of the block with the CCD camera, as shown in Fig. 2 (right). Patients underwent simultaneous RPM and bellows 4D CT acquisition during their initial CT simulation using the same procedures outlined in the phantom experiment. 4D ${\text{CT}}_{\text{BELLOWS}}$, 4D ${\text{CT}}_{\text{RPM}},{\text{MIP}}_{\text{BELLOWS}}$, and ${\text{MIP}}_{\text{RPM}}$ were generated. Bellows and RPM waveforms were exported from the research EBW and RPM workstation, respectively, for subsequent analysis.

**Table 2:** Patient demographics for external surrogate evaluation study

*Patient*	*Gender*	*Staging*	*Patient Age (Yrs)*	*PTV Size (CC)*	*Tumor Location*
1	F	T1N0M1	82	11.48	LUL
2	M	T2N1M0	76	418.94	LUL
3	F	T3N3M0	68	528.13	RUL
4	F	T2NXM1	80	481.87	LUL
5	F	T4NXM1	78	200.29	RUL
6	M	Unspecified	42	146.04	RLL
7	M	TI	74	36.95	RUL
8	F	T4	61	23.51	LUL
9	M	T2N1M0	68	253.45	RML
10	M	T2N0M0	82	243.94	LUL

F = Female; M = Male; LUL = left upper lobe; RUL = right upper lobe; RLL = right lower lobe; RML = right middle lobe.

### E. Waveform analysis

Because the experimental configuration detected periods of X‐RAY ON for both bellows and RPM, the start of 4D CT acquisition was set as the origin (i.e., zero time point) for all waveforms, and served as a mutual reference point between external surrogates. All waveform analyses were performed only during the periods of X‐RAY ON that corresponded to 4D CT data. Due to differences in sampling frequency, bellows waveforms (~38Hz) were downsampled in the time domain to match RPM (30 Hz) acquisition (OriginLab, version 6.1, OriginLab Corporation, Northampton, MA). Bellows amplitude displacement was in arbitrary units (pressure differential), whereas the RPM reports displacement in mm. Therefore, to facilitate cross‐comparison among the external surrogates, bellows and RPM waveforms were normalized to the maximum waveform peak value and comparisons were made between normalized data. The peaks of the displacement‐time curves were determined by finding the local maximum displacements via a bounding box and search function. In curves with no distinct peaks (e.g., truncated or plateau features), midpoints in the peaks' flattened region were used. Absolute time differences between the calculated end‐inhale peak times from the bellows and RPM waveforms were computed to characterize the latency difference between the systems (i.e., RPM end‐inhale peaks less the bellows end‐inhale peaks, with a positive value indicating that RPM end‐inhale occurred before bellows end‐inhale). Frequency histograms of the latency were plotted and assessed.

Relative amplitude differences were calculated and compared for each breathing trace, with a Pearson correlation coefficient testing for agreement. Coefficients of variation (COV) of the amplitude, defined as a ratio between the standard deviation and the mean, were calculated to characterize variability in breathing amplitudes between the RPM and bellows systems. COV describes the dispersion of a probability distribution and has been used to characterize inter‐ and intra‐observer contouring variability.^(^ ^12^ ^,^ ^13^ ^)^ Differences between RPM and bellows metrics were assessed via nonparametric Mann‐Whitney *U* tests.

### F. Image analysis

All phantom and patient 4D CT image data and MIPs were imported into Eclipse Treatment Planning System (Varian Medical Systems, v10.0) via DICOM filters for subsequent image analysis and processing.

#### F.1 Phantom study

A delineation study was conducted for all phantom data, with the same lung window/level used for all contouring. To eliminate the potential for interobserver variability, a single physicist performed all of the delineations. A volume of interest was selected around the target object, and auto‐thresholding was performed with values of about −900to−600HU. Manual adjustment was performed on each contour as deemed necessary. Internal target volumes (ITVs) were delineated on the ${\text{MIP}}_{\text{BELLOWS}}$ and ${\text{MIP}}_{\text{RPM}}$ images for all phantom cases to yield the ${\text{ITV}}_{\text{BELLOWS}}$ and ${\text{ITV}}_{\text{RPM}}$, respectively. The corresponding 4D CTs were reviewed for consistency between the delineated ITVs and object of interest. Image difference maps $\left({\text{MIP}}_{\text{BELLOWS}}-{\text{MIP}}_{\text{RPM}}\right)$ were generated to evaluate local density changes, and individual 4D CT phases were evaluated for congruence.

RPM and bellows contour similarity was assessed through percent volume change, centroid location, Dice similarity coefficient (DSC),^(14)^ and overlap index (OI). For all phantom cases studied, ${\text{ITV}}_{\text{BELLOWS}}$ was considered the “accepted” because these data were sorted on the clinical workstation using the CT configuration at the time of image acquisition. Percent volume change was calculated by subtracting the ${\text{ITV}}_{\text{RPM}}$ from the ${\text{ITV}}_{\text{BELLOWS}}$, dividing by ${\text{ITV}}_{\text{BELLOWS}}$, and multiplying by 100%. DSC was defined by Eq. (1):

(1) $$\text{DSC}=2({\text{ITV}}_{\text{BELLOWS}}\cap {\text{ITV}}_{\text{RPM}})/({\text{ITV}}_{\text{BELLOWS}}+{\text{ITV}}_{\text{RPM}})$$

DSC is a spatial overlap index that ranges from 0 to 1, where 0 indicates spatial overlap between two segmentation results while the latter indicates complete overlap.^(15)^ The OI was defined as the ratio of the mutual volume to the treated volume as described by Tsuji et al.^(16)^ and shown in Eq. (2):

(2) $$\text{OI}=({\text{ITV}}_{\text{BELLOWS}}\cap {\text{ITV}}_{\text{RPM}})/{\text{ITV}}_{\text{BELLOWS}}$$

The DSC serves as a measure of the similarity between the tested volumes, while the OI represents the inclusion of ${\text{ITV}}_{\text{BELLOWS}}$ within ${\text{ITV}}_{\text{RPM}}$.^(16)^ DSC has been successfully employed for comparing segmentations of prostate gland peripheral zones in magnetic resonance image (MRI) guidance for brachytherapy,^(^ ^15^ ^,^ ^17^ ^)^ evaluating automatic delineation on computed tomography scans for head and neck cancer cases,^16^, ^18^ and analyzing segmentation agreement of white matter lesions in MRI.^(15)^ Changes in overall target position were quantified by calculating the center of mass differences between the ${\text{ITV}}_{\text{RPM}}$ and ${\text{ITV}}_{\text{BELLOWS}}$.

Comparisons were also made to “ground truth” phantom internal target volumes (${\text{ITV}}_{\text{TRUE}}$) for each breathing curve calculated by subtracting the experimental ITV (i.e., ${\text{ITV}}_{\text{RPM}}$ and ${\text{ITV}}_{\text{BELLOWS}}$) from the ${\text{ITV}}_{\text{TRUE}}$, dividing by ${\text{ITV}}_{\text{TRUE}}$, and multiplying by 100%. ${\text{ITV}}_{\text{TRUE}}$ comparisons were made because they represent the phantom volume in the absence of 4D CT sorting artifacts and interplay effects. Statistical associations were assessed via Pearson correlation coefficients.

### F.2 Patient study

For the patient study, no ground truth data were available. Recently, Louie et al.^(12)^ demonstrated that considerable delineation variability exists when contouring patients' lung tumors in 4D CT, largely due to 4D CT artifacts, atelectasis, and nearby vessels that may confound tumor boundaries. For these reasons, patients' tumor volumes were not contoured in the same manner as the phantom analysis. For all patient cases, to reduce inter‐ and intra‐observer variability, automatic lung segmentation was performed on the RPM and bellows MIP images in Eclipse Treatment Planning System (v11.0; Varian Medical Systems). Briefly, the automated segmentation tool identifies the CT slice with the largest amount of air in two connected areas that are required to originate from inside the external body structure. A flood fill algorithm, iteratively filling the lung volume, is then applied.^(19)^ Segmentation results were reviewed by an experienced physicist, and manually modified, if deemed necessary. Percent volume change, DSC, and OI were calculated as defined for the phantom data. In addition, image difference maps $\left({\text{MIP}}_{\text{BELLOWS}}-{\text{MIP}}_{\text{RPM}}\right)$ were generated in ImageJ (available at: http://rsb.info.nih.gov/ij/download.html)^(20)^ to elucidate changes in image intensity. Individual 4D CT phases were reviewed and differences were evaluated.

## III. RESULTS

### A. Phantom results

**Figure 3:**
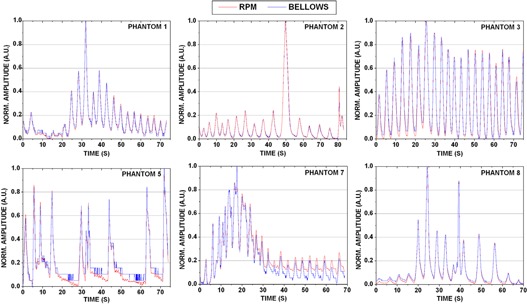
Phantom breathing traces obtained using Varian's RPM and Philips' bellows pneumatic belt for six different programmed breathing curves derived from patient data. Strong agreement was observed between breathing traces for all eight phantom cases studied

**Figure 4:**
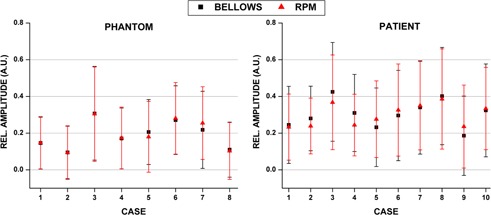
Mean and standard deviation of the amplitudes for waveforms generated via simultaneous bellows and RPM acquisition for eight irregular breathing patterns in eight phantom waveforms (left) and prospective patient study (n=10) (right).

Table 1 summarizes the phantom breathing curve statistics, including breathing rate, amplitude, and coefficient of variation. Near perfect correlation was observed among all eight breathing traces examined (Pearson′sr=0.994±0.005). Figure 3 illustrates the agreement for six of the eight curves studied for bellows and RPM waveforms. Slight discrepancies were observed with low amplitude displacements (Fig. 3, Phantoms 2 and 5). In some instances, the bellows was found to truncate the very extreme signal positions (e.g., Fig. 3 Phantom 5, 20–30 sec), although this did not impact the overall agreement between the bellows and RPM nor did it impact image quality. Figure 4 (left) demonstrates the mean normalized amplitude for each phantom breathing curve obtained with the RPM and bellows over the X‐RAY ON region. The largest amplitude difference between external surrogates was calculated for Phantom 7 (bellows was 0.04 A.U. less than RPM), where the RPM deviated slightly from the bellows at low amplitude displacements. This case is illustrated in Fig. 3, where RPM and bellows amplitudes were well matched until ~30sec into the scan, and then deviations were observed as the breathing curve amplitude reduced.

**Figure 5:**
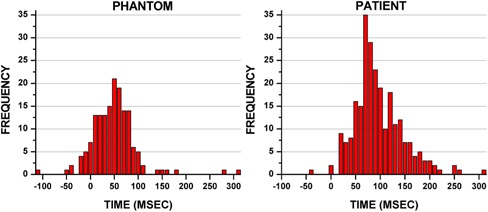
Absolute time differences between the calculated end‐inhale peak times from the bellows and RPM waveforms calculated to characterize the latency difference between the systems (i.e., RPM end‐inhale peaks less the bellows end‐inhale peaks, with a positive value indicating that RPM end‐inhale occurred before bellows end‐inhale).

The population histogram distribution summarizing the absolute time difference (i.e., latency) between the end‐inhale peaks for the RPM and bellows waveforms in the phantom is shown in Fig. 5 (left). The mean time difference between external surrogates was 25.6±38.3ms(range:−119to155ms), with the RPM typically leading the bellows. Table 3 summarizes the phantom contouring results. Over all, the phantom targets contoured on the MIP images, the volumes were 200.36±11.9cc and 199.83±12.6cc for the RPM and bellows targets, respectively, and were not significantly different (U=33,p>0.05). Only slight positional variations in the centers of mass were observed between the bellows and RPM contoured MIP targets: 0.025mm±0.05mm,0.013±0.04mm,and−0.24±0.31mm in lateral (LAT), anterior‐posterior (A‐P), and superior‐inferior (S‐I) directions, respectively. The largest volume difference was observed for a case where the RPM volume was 3.6 cc or 1.77% larger than the delineated bellows target volume (Phantom 1). The corresponding waveform for this case is shown in Fig. 3, top left, Phantom 1. Bellows and RPM ITVs yielded strong agreement and near perfect concordance for both DSC and OI (0.96±0.00 and 0.96±0.01, respectively).

**Table 3:** Volume and positional changes due to differences in delineated phantom internal target volumes generated from bellows and RPM. Differences from ${\text{ITV}}_{\text{TRUE}}$ are also provided

*Phantom Case*	*Volume Change (%)*	*Overlap Index (A.U.)*	*Dice Similarity (A.U.)*	*Centroid Differences (X*, *Y*, *Z*, *mm*)	${\text{ITV}}_{\text{TRUE},\;\text{Volume}}\;{\text{ITV}}_{\text{BELLOWS Change}}\;(\%)$	${\text{ITV}}_{\text{TRUE},\;\text{Volume}}\;{\text{ITV}}_{\text{RPM Change}}\;(\%)$
1	−1.77	0.974	0.965	(0.1,0.1,−0.1)	16.67	15.19
2	−1.25	0.971	0.965	(0.0,0.0,−0.2)	25.12	24.18
3	0.46	0.964	0.966	(0.1,0.0,−0.1)	2.08	2.53
4	0.61	0.959	0.962	(0.0,0.0,−0.2)	12.10	12.64
5	−1.21	0.967	0.961	(0.0,0.0,−0.3)	15.98	14.97
6	−0.70	0.968	0.964	(0.0,0.0,−0.2)	9.73	9.09
7	0.40	0.957	0.959	(0.0,0.0,−0.3)	6.90	7.27
8	1.15	0.957	0.963	(0.0,0.0,−0.9)	12.21	13.22
Population				(0.03±0.05,		
Mean±	−0.29±1.07	0.96±0.01	0.96±0.00	0.01±0.04,	12.60±6.93	12.39±6.42
StDev				−0.24±0.31)		

When compared to the ${\text{ITV}}_{\text{TRUE}}$, a volume generated via delineating the phantom object paused at inhale and exhale motion platform positions, the ${\text{ITV}}_{\text{RPM}}$ and ${\text{ITV}}_{\text{BELLOWS}}$ underestimated the volume an average of ~12%. Across the eight phantom breathing curves, similar deviations from the “ground truth” volumes were observed for both external surrogates (range: 2.53%‐24.18% difference for ${\text{ITV}}_{\text{RPM}}$ and 2.08%‐25.12% difference for ${\text{ITV}}_{\text{BELLOWS}}$). A strong, statistically significant positive association was observed between the percent difference from ${\text{ITV}}_{\text{true}}$ and CV for RPM (Pearson's r=0.74,p<0.05. However, the association between ${\text{ITV}}_{\text{true}}$ and CV for bellows was not statistically significant (Pearson's r=0.63,p=0.1).

### B. Patient results

Figure 4 (right) summarizes the latency distribution between the end‐inhale peaks for the RPM and bellows waveforms, with a mean difference of 79.7±45.5ms(range:−47to242ms). Like the phantom results, the RPM data led the bellows data. Table 4 illustrates the patient breathing curve statistics for simultaneous RPM and bellows waveform acquisition. The patient cases showed similar distributions of varying amplitudes and breathing rates as the phantom experiments. Differences between the normalized amplitudes of the RPM and bellows data were not statistically significant. The largest amplitude difference occurred for case 7 (bellows was 0.03 A.U. less than RPM), although bellows amplitudes were slightly lower than RPM for six of eight phantom cases studied.

**Table 4:** Patient breathing curve and lung volume statistics for waveforms acquired simultaneously using RPM and bellows

*Patient*	*External Surrogate*	Amplitude Mean±StDev(A.U.)	*Amplitude %CV*	*Breathing Rate (BPM)*	*Pearson's r* ^a^	*Volume Change (%)*	*Overlap Index (A.U.)*	*Dice Similarity (A.U.)*
1	Bellows	0.245±0.210	85.6	9.6	0.934	−0.17	0.997	0.996
	RPM	0.233±0.180	77.1					
2	Bellows	0.280±0.176	62.8	16.9	0.974	0	0.996	0.996
	RPM	0.239±0.152	63.8					
3	Bellows	0.425±0.269	63.3	22.3	0.881	0.10	0.992	0.993
	RPM	0.368±0.258	70.1					
4	Bellows	0.310±0.210	67.9	23.2	0.908	0.08	0.991	0.992
	RPM	0.244±0.168	69					
5	Bellows	0.232±0.214	92.1	12.6	0.969	0.06	0.997	0.997
	RPM	0.276±0.209	75.8					
6	Bellows	0.296±0.246	83.2	13.6	0.968	−0.17	0.993	0.992
	RPM	0.326±0.251	77					
7	Bellows	0.340±0.254	74.7	18.6	0.985	−1.23	0.995	0.989
	RPM	0.350±0.241	69					
8	Bellows	0.402±0.265	65.9	14.2	0.977	0.07	0.993	0.993
	RPM	0.386±0.273	70.7					
9	Bellows	0.186±0.216	115.7	7.0	0.967	−0.50	0.990	0.988
	RPM	0.236±0.226	95.6					
10	Bellows	0.324±0.253	78.2	21.8	0.911	−1.17	0.992	0.986
	RPM	0.334±0.224	67.3					
Population								
Mean±						−0.293±	0.994±	0.993±
Stdev						0.512	0.002	0.004

a All values were statistically significant (p < 0.0001).

**Figure 6:**
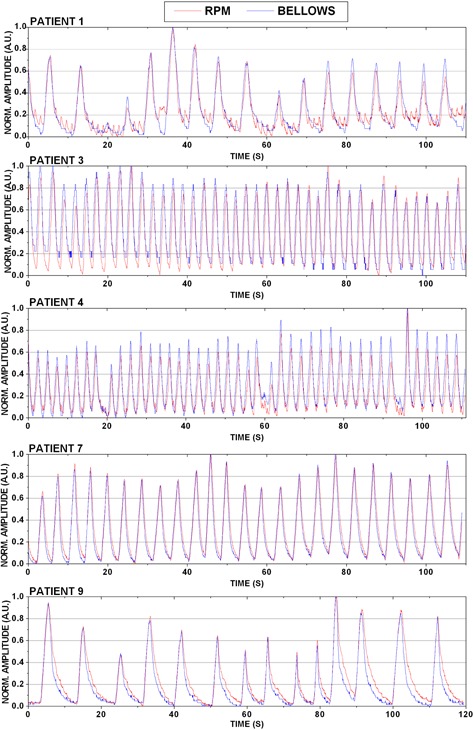
Patient external waveforms obtained using Varian's RPM and Philips' bellows pneumatic belt for five different patients. Detailed descriptions are given in text.

While strong agreement was observed among all patient RPM and bellows breathing curves (Pearson′sr=0.881−0.985) the results were not as well correlated as the phantom data. Figure 6 demonstrates a subset of breathing curves for five of the ten patients studied. For Patient 1, RPM revealed slight reverberations in the abdomen near end‐exhale that were undetected by the bellows. Patient 4 demonstrated this same phenomenon, particularly at end‐exhale portions of the RPM waveform. Patient 3 had the worst association between the RPM and bellows data of all ten patients studied (Pearson's r~0.88). For this patient, discrepancies in the breathing curves can be observed in Fig. 6, particularly in the first ~50sec of data collection. Here, the RPM peaks were skewed compared to the bellows peaks, leading to a systematic offset in the end‐inhale peak values. On average, RPM end‐inhale peaks were ~117ms ahead of bellows for this particular patient. Patient 7 demonstrated strong, periodic breathing patterns with nearperfect correlation between waveforms (Pearson's r~0.985). Some differences in the transition phase to exhale were observed for Patient 9, where the curves closely matched on inhale and deviated on exhale.

Table 4 summarizes the near‐perfect agreement between the lung contours on ${\text{MIP}}_{\text{RPM}}$ and ${\text{MIP}}_{\text{BELLOWS}}$ (OI and DSC were near 1.00). The largest percent difference between RPM and bellows lung volumes was for patient 7, with the bellows lung volume ~1% smaller (39 cc) than the RPM lung volume. Even with the slight difference in volume, high OI and DSC values suggested strong concordance between volumes. To evaluate the dosimetric impact of the volume difference, both RPM and lung volumes were mapped to the clinically used treatment plan. The mean lung dose varied only 1.6 cGy between volumes, and $V_{20}$ (i.e., percentage of lung volume receiving 20 Gy) differed by 0.03%. Image quality differences were also assessed via subtraction images between the ${\text{MIP}}_{\text{RPM}}$ from the ${\text{MIP}}_{\text{BELLOWS}}$. In general, no appreciable differences were observed between MIP difference maps based on waveform origin. Example images for Patient 3, who demonstrated the worst association between waveforms, are shown in Fig. 6. Here, difference maps shown between MIP, end‐inhale, and end‐exhale images showed negligible differences. Similar results were observed for individual phases (not shown).

## IV. DISCUSSION

This study sought to compare two commonly used external surrogates (RPM and bellows) acquired simultaneously for 4D CT sorting. Breathing curves, CT images, and phantom target delineation were assessed. Overall, breathing curves obtained using both RPM and bellows simultaneously were found to have near‐perfect association for all phantom cases studied. Slight disagreement was observed with low amplitude displacements, particularly for Phantom curve 5 (Fig. 3, bottom right) near the end‐exhale portions of the breathing curves (i.e., between 20–30 sec, 30–45 sec, and 50–60 sec). Here, RPM was able to detect slight downward drifts, while the bellows signal remained nearly constant. In this case, however, the midpoint transitions and end‐inhale peaks were well matched. This suggests that the bellows belt may not have been taut enough to detect the slight signals when the phantom motion platform was at its lowest displacement. This effect was not observed for any other phantom cases studied. Furthermore, these results did not translate into delineation error in 4D CT image analysis, as no significant differences were observed between the phantom target volumes contoured on the MIP images or in the centroid position of the targets. 4D CT reconstruction artifacts were also negligible in the phantom. These results were as expected due to rigid phantom geometry translating the RPM block and bellows belt in an identical fashion.

An average of ~12% underestimation in phantom volume was found when comparing ${\text{ITV}}_{\text{TRUE}}$ to both ${\text{ITV}}_{\text{BELLOWS}}$ and ${\text{ITV}}_{\text{RPM}}$. Phantom cases with higher variability (i.e., higher coefficients of variation) tended to have a higher volume discrepancy from ${\text{ITV}}_{\text{TRUE}}$. This result was consistent with the literature. For example, in a 4D CT phantom study of simulated patient breathing curves conducted by Park et al.,^(21)^ MIPs generated from 4D CTs were systematically less than expected. Another observation was that phantom curves with lower breathing rate (i.e., fewer cycles during the measured timeframe) tended to have worse volume agreement with ${\text{ITV}}_{\text{true}}$ for both RPM and bellows (Pearson′sr~−0.60), although not statistically significant (p~0.11). This also agrees with what has been reported in the literature.^(21)^

For the prospective study of ten patient cases, strong associations between RPM and bellows waveforms were observed (mean Pearson′sr=0.947±0.037), although these were not as strong as observed in the phantom. This can be expected, however, because the motion platform was translated with fixed, rigid geometry for both the bellows and RPM block. Whereas for the patient, the bellows and RPM block could not be placed in the same exact location on the abdomen, as demonstrated in Figs. 2 (middle) and (right). It has been shown that the respiration signal can vary depending on the placement on the abdomen,^(22)^ so it can be expected that the input signals would not be identical between the RPM and bellows. Nevertheless, when comparing motion platform measurements for RPM and a pressure sensor for gated delivery, Li and colleagues^(23)^ found 98.2%‐99.6% agreement, which was consistent with our phantom findings. Our results also agreed with a study of ten patients by Otani et al.,^(9)^ who found strong correlations between RPM and a small pressure sensor (Pearson′sr=0.940−0.994). The pressure sensor studied by Otani et al. involves a small pressure sensor inserted into the pocket of a belt, which was not the same pneumatic system studied in this work. Spadea et al.^(24)^ and Kauweloa et al.^(25)^ explored the differences between using surface imaging cameras (GateCT, VisionRT Ltd., London, UK) to track the patient's abdomen as an external surrogate with Varian's RPM system in a variety of 4D CT phantom experiments. Like our study, both investigators obtained near‐perfect correlations between GateCT and RPM with phantom measurements. However, Kauweloa's work revealed that for 12 patient cases, correlations in external surrogates ranged from 0.724 to 0.985. The association strength was similar in our study, although this group did not assess the impact of surrogate on 4D CT image quality.

Because of the differences between units in the RPM and bellows system (i.e., mm and pressure differential, respectively), waveform absolute displacement analysis was not possible. However, relative amplitudes could be computed and these were not statistically significant between systems. This agrees with results observed by others comparing different external surrogates.^(9)^ It was noted that in some cases, the bellows signal appeared to plateau in periods of small displacement or near end‐exhale. This can be observed in the phantom (Fig. 3, Phantom 5) and patient (Fig. 6, Patient 1). The patient example here showed some additional motion that was detected by RPM but not by bellows. In the patient, a possible cause of this was the block being placed on an uneven region of the abdomen thereby causing the block itself to have irregular input signal, or the RPM picking up some cardiac signal. Even with these differences, the MIPs and ten phases of the 4D CT were reviewed by experienced physicists and were felt to have no detectable differences in 4D CT sorting quality.

**Figure 7:**
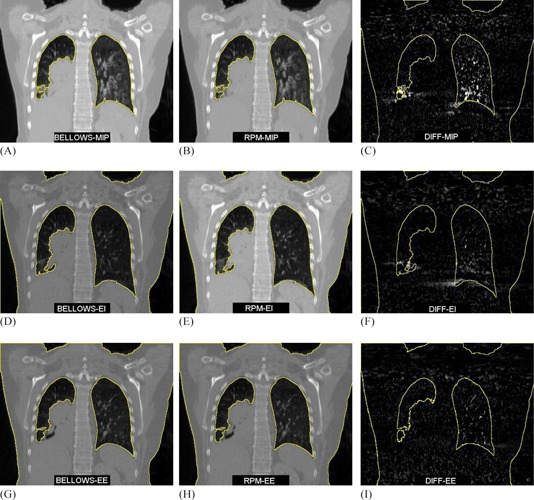
Coronal images for Patient 3, who had the worst waveform association between RPM and bellows. MIP Bellows, MIP RPM, and difference map (i.e., subtraction of RPM image from bellows image) are shown in (A)‐(C), while end‐inhale (EI, 0%) and end‐exhale (EE, 50%) are demonstrated in (D)‐(F) and (G)‐(I), respectively. All contours were outlined on the bellows images and overlaid on the RPM and difference map images to assist in image assessment.

On average, percent difference in delineated patient lung volumes was −0.30%±0.52%(range:−1.23%to0.10%) between RPM and bellows reconstructions. Both DSC and OI yielded strong agreement between the lung volumes, as shown in Table 4. This suggests that differences in the external surrogate waveform may not necessarily result in delineation differences. This is further supported by Fig. 7 that demonstrates CT reconstructions images for patient with the lowest strength of waveform association (Patient 3). Here, none of the images were sensitive to external surrogate (i.e., the difference map was virtually zero), including the MIP, end‐inhale, and end‐exhale phases. Review of the patient's experimental setup (see Fig. 2, right) shows that the bellows may have been affected by the presence of the ribcage, and it can be postulated that this could have caused the shallower excursion (i.e., distance between end‐inhale and end‐exhale) for the bellows during the first ~50sec of acquisition (Fig. 6, Patient 3). Even with the lowest association between waveforms, however, little difference was observed between the 4D CT datasets.

The latency, or absolute time difference, between the RPM and bellows end‐inhale peaks was found to be <100ms between the two systems for both phantom and patient data. The histogram data shown in Fig. 5 demonstrates that for most of the end‐inhale peaks studied in phantom and patient, the latency (RPM end‐inhale time point less the bellows end‐inhale time point) was greater than 0. This suggests that RPM data led the bellows data slightly, although this may have been sensitive to the peak‐picking functionality used in this work. These results are similar to a recent comparison between peak times of RPM less the peak times for a pressuresensitive sensor in patients, where the time differences ranged from −520to600msec, with an overall average of all peaks of 24 msec.^(9)^

Because ROI analysis revealed negligible differences in MIP target volumes for the phantom study and lung volumes in the patient study, only cursory dosimetric analysis was explored in this work for the patient with the largest volume differences between RPM and bellows reconstructions. Negligible, clinically insignificant differences in lung dose were observed when RPM and bellows lung volumes were used for Patient 7. This study sorted data into 10 4D CT phases to elucidate slight changes arising from the selection of external surrogate, although similar target volumes have been reported between delineations performed on 10 phases and those contoured using 4 and 6 phases.^(^ ^26^ ^,^ ^27^ ^)^ Thus it can be postulated that comparable results would be obtained with fewer phases.

Another limitation of this study is that the waveform analysis required rescaling the breathing curves obtained from RPM and bellows. While these two breathing signals were found to be highly correlated, it is possible that the amplitudes could vary between the two systems. Future work may involve incorporating an amplitude‐based binning technique for raw data reconstruction of 4D CT phases to better elucidate these effects, as described recently.^(25)^ In this study, 4D CT sorting with Varian RPM was performed off‐line using a research EBW that allowed loading an additional waveform for reconstruction. While this added functionality was experimental, the raw data, reconstruction algorithm, Varian RPM acquisition, and subsequent VXP file generation were identical to clinically released software and hardware, and thus not expected to influence the results.

Practically speaking, each mode of external surrogate acquisition has its advantages and disadvantages. For example, the bellows system offers a simpler configuration that comes with the purchase of 4D CT functionality in Philips Big Bore systems (i.e., more of a “plug and play” application) at the expense of not being translatable to gating at the linear accelerator. The bellows is made of a latex rubber material, requiring proper handling for patients with latex allergies, and should be inspected for cracks and tears in the rubber that can degrade performance over time due to constant clinical use. RPM, on the other hand, requires the additional expense of the hardware/software, but with the added benefit of potentially gating radiation therapy treatments. RPM requires mutual drive mapping between both the CT scanner and the RPM software to export the RPM waveform, which has, at times, complicated instances of 4D CT acquisition at our institution. The RPM marker blocks can also be sensitive to cracks in a clinical environment and should be checked periodically for performance. While each institution should weigh benefits of external surrogate selection against its own clinical needs, this study has revealed that comparable, and nearly equivalent, results can be found with both the RPM and bellows systems.

## V. CONCLUSIONS

Slight differences were observed in waveform and latency analysis between Philips bellows and Varian's RPM, although these did not translate to differences in image quality or impact delineations. Therefore, the two systems were found to be equivalent external surrogates in the context of 4D CT for treatment planning purposes.

## ACKNOWLEDGMENTS

This work is supported in part by Philips Medical Systems. The authors would like to thank Paul Klahr at Philips for his technical expertise and support related to the research configuration of the research EBW, and Jonathon Reinhart for his efforts during preliminary data collection.
